# 1173. Risk of Pneumococcal Disease and Rate of Pneumococcal Vaccination Among Medicare Beneficiaries Aged 65 Years and Older Residing in Long-Term Care Facilities — United States, September 1, 2014 – December 31, 2019

**DOI:** 10.1093/ofid/ofad500.1013

**Published:** 2023-11-27

**Authors:** Miwako Kobyashi, Kristin Andrejko, Lindsay Zielinski, Arnstein Lindaas, Hyejeong Cha, Bradley Lufkin, Yoganand Chillarige

**Affiliations:** CDC, Atlanta, Georgia; CDC, Atlanta, Georgia; CDC, Atlanta, Georgia; Acumen LLC, Burlingame, California; Acumen, LLC, San Francisco, California; Acumen, LLC, San Francisco, California; Acumen LLC, Burlingame, California

## Abstract

**Background:**

Adults living in long-term care (LTC) facilities are at increased risk of respiratory infectious diseases, including pneumococcal disease. On August 13, 2014, the Advisory Committee on Immunization Practices recommended 13-valent pneumococcal conjugate vaccine (PCV13) in addition to 23-valent pneumococcal polysaccharide vaccine (PPSV23) for adults aged ≥65 years based on data that showed PCV13 vaccination confers protection against all-cause community-acquired pneumonia (CAP) and invasive pneumococcal disease (IPD). We assessed PCV13 vaccination coverage and incidence of CAP and IPD among Medicare beneficiaries with ≥1 LTC stay.

**Methods:**

We identified Medicare Fee-for-Service beneficiaries aged ≥65 years with no prior PCV13 receipt and ≥1 LTC facility stay during September 1, 2014 through December 31, 2019 through Minimum Data Set assessments and skilled nursing facility claims. PCV13 and PPSV23 vaccination history was determined through Medicare claims using Healthcare Common Procedure Coding System and Current Procedural Terminology codes. Incident IPD and CAP outcomes were identified through inpatient Medicare claims using International Classification of Diseases diagnosis codes (ICD-9-CM/ICD-10-CM). Outcome incidence was calculated based on time during and outside LTC stays. PCV13 coverage was assessed by LTC residency status on December 31, 2019.

**Results:**

There were 3,643,248 beneficiaries with ≥1 LTC facility stay during the observation period. Beneficiaries were majority white (89.9%), female (62.1%), aged ≥75 years (67.7%), and had ≥1 chronic medical condition (55.7%); 25.6% had received PPSV23 vaccination at the study start date. By December 31, 2019, PCV13 coverage was 55.3%; coverage among those residing in and outside a LTC facility was 47.5% and 57.3%, respectively. Incidence of CAP and IPD were higher during LTC compared with time outside LTC stay (incidence rate ratio 3.17 [95% CI: 3.15, 3.19] and 1.90 [95% CI: 1.80, 2.00] for CAP and IPD, respectively) (Table).

**Figure:**
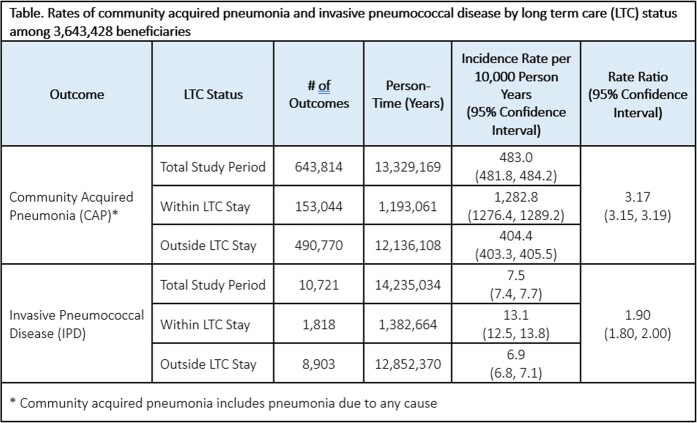

**Conclusion:**

Incidence of IPD and CAP were higher during an LTC stay compared with time residing outside of a LTC facility. PCV13 coverage was lower among those in LTC. Improving pneumococcal vaccination coverage may help mitigate the risk of IPD and CAP in this population.

**Disclosures:**

**All Authors**: No reported disclosures

